# China Multi-Center Preschool Autism Project (CMPAP): Design and Methodologies to Identify Clinical Symptom Features and Biomarkers of Autism Spectrum Disorders

**DOI:** 10.3389/fpsyt.2020.613519

**Published:** 2021-02-09

**Authors:** Ting Yang, Jiang Zhu, Qiu Li, Li Chen, Li-Jie Wu, Fei-Yong Jia, Yan Hao, Xiao-Yan Ke, Ming-Ji Yi, Chun-Hua Jin, Jie Chen, Ting Yu Li

**Affiliations:** ^1^Children's Nutrition Research Center, Children's Hospital of Chongqing Medical University, Chongqing Key Laboratory of Childhood Nutrition and Health, Ministry of Education Key Laboratory of Child Development and Disorders, National Clinical Research Center for Child Health and Disorders, China International Science and Technology Cooperation Base of Child Development and Critical Disorders, Chongqing, China; ^2^Department of Child and Adolescent Health, School of Public Health, China Medical University, Shenyang, China; ^3^Department of Rehabilitation Medicine, The First Hospital of Jilin University, Changchun, China; ^4^Department of Pediatrics, Tongji Hospital, Tongji Medical College, Huazhong University of Science and Technology, Wuhan, China; ^5^Nanjing Brain Hospital Affiliated to Nanjing Medical University, Nanjing, China; ^6^Affiliated Hospital of Qingdao University, Qingdao, China; ^7^Department of Child Health Care, Capital Institute of Pediatrics, Beijing, China

**Keywords:** autism spectrum disorders, biomarkers, clinical symptoms, ICD-11, early diagnosis (MeSH)

## Abstract

**Background:** The etiology of autism spectrum disorder (ASD) has not yet been fully identified, but it seems to be triggered by complex genetic and environmental risk factors. Moreover, the tremendous etiological and clinical differences among individuals with ASD has had a major negative impact on early diagnosis and individualized treatment. Earlier diagnosis of precise clinical subtypes of ASD could lead to individualized treatment and a better prognosis. However, few large-scale epidemiological studies have explored precise clinical subtypes and clinically meaningful biomarkers, especially in China.

**Methods and Design:** The China Multi-center Preschool Autism Project (CMPAP) includes nearly 3,000 children−1,469 individuals with ASD and 1,499 typically-developing (TD) controls—from 13 cities in China. Using a case-control design, each participant was comprehensively characterized in terms of feeding and disease history, maternal history, family history, clinical core symptoms, comorbidities, biochemical markers, genomics, urine/fecal metabonomics, and intestinal flora. In addition, data on environmental risk factors were obtained using interviews and electronic medical records.

**Conclusion:** The study was designed to: (1) investigate age at diagnosis and treatment and family and social support for preschool children with ASD in China, (2) develop a more accurate clinical subtype and intervention system for the ICD-11, and (3) find the specific genes and environmental markers of different subtypes, which will help in the development of early diagnosis and individual intervention programs for preschool children with ASD. This study will provide the basis for improving national health policies for ASD in China.

## Introduction

Autism spectrum disorder (ASD) is a common neurodevelopmental disorder that is characterized by social and communication impairments, restricted interests, and repetitive behaviors (1). Approximately 18.5 per 1,000 children of 8 year-old children in the USA have ASD (2), and the ASD prevalence rate in China has been estimated to be 0.7% among children who are 6 to 12 years old (3). Moreover, ASD is the most common mental disability in China, according to national census data on individuals with disabilities (4); hence, the diagnosis and treatment of ASD has gained increasing attention in China. The guidelines of the American Academy of Pediatrics (AAP) state that early diagnosis, early intervention, social and family support, intelligence (e.g., IQ), and language ability all play key roles in the rehabilitation of children with ASD (1). Yet, no large-scale, multi-center epidemiological surveys have been conducted in China, to date, to determine the etiology of ASD in children, their age at diagnosis and treatment, their characteristics and clinical symptoms, or the effects of social and family support on their ASD symptoms, using precise clinical subtypes.

Clinically, the severity of core symptoms and comorbidities vary greatly among individuals with ASD, but gastrointestinal disturbances, sleep disorders, and poor eating habits are common comorbidities in children. Unfortunately, most pediatricians in China do not pay enough attention to the comorbidities of ASD, and the incidence of comorbidities in children with ASD and the relationship between comorbidities and core ASD symptoms are still unknown. A variety of clinical symptoms and environmental and genetic risk factors are widely recognized among individuals with ASD, but none of the susceptible genes or environmental risk factors seem to be shared by all individuals with ASD (5). This heterogeneity has been a major obstacle to the development of effective treatments (6). Therefore, it is crucial to identify clinical subgroups and find meaningful biomarkers (7). At present, there is no standard classification of ASD in children, and no specific biomarkers have be identified.

Most studies, to date, have used small single-center samples and case-control designs to look for biomarkers, such as functional magnetic resonance imaging (fMRI), eye tracking (8, 9), or markers of oxidative stress and DNA methylation (10). However, ASD involves a variety of pathophysiological mechanisms and universal biomarkers may not exist for all the subtypes of ASD (11). Furthermore, many studies have focused on certain measures, such as scores on a scale (e.g., the Childhood Autism Rating Scale, Autism Diagnostic Observation Schedule, Autism Diagnostic Interview-Revised) or some statistical model to classify ASD children. Due to their small sample sizes and the limitations of their research methods, these studies may have overlooked the classification of comorbidities and failed to divide children with ASD into clinically meaningful subgroups. Thus, it is impossible to find the specific biomarkers of each subgroup. The International Classification of Diseases, Eleventh Revision (ICD-11) divides ASD into 6 subtypes based on levels of intelligence and language impairment, which is conducive to more accurate clinical interventions. Therefore, identifying common comorbidities of children with ASD, using the ICD-11 for precise clinical typing, will be helpful for etiological research on ASD. To our knowledge, there is no study of ASD subtypes based on the ICD-11 and common comorbidities.

Hundreds of genes have been identified in individuals with ASD (12), and it has been estimated that the heritability of ASD is approximately 80% (13). On the other hand, a number of systematic reviews and meta-analyses have described environmental risk factors (e.g., prenatal and perinatal factors and maternal dietary and lifestyle factors) that play an important role in the development of ASD. However, none of the susceptible genes or environmental risk factors seem to be shared by all individuals with ASD (5), and that heterogeneity has been a major obstacle to the development of effective treatments. Therefore, it is crucial to identify clinical subgroups and find meaningful biomarkers.

In summary: (1) there is no standard classification of ASD in children; (2) no specific biomarkers have been identified; (3) as ASD involves a variety of pathophysiological mechanisms, a universal biomarker of all its subtypes may not exist (11); and (4) given their small sample sizes and methodological limitations, studies have failed to divide children with ASD into meaningful subgroups. Therefore, it has not been possible to find the specific biomarkers of each subgroup.

Hence, it is necessary to investigate ASD in preschool children in China, including their age at diagnosis and treatment, their common comorbidities, and the effects of family and social support on their symptoms, and to identify clinically useful biomarkers based on clinical subtypes of the ICD-11. This requires a large-scale multi-center study, using a unified standard, rigorous research procedures, and sophisticated statistical analyses. We received support from the General Program of the National Natural Science Foundation of China (Nos. 81771223 and 81770526), the Guangzhou Key Project in “Early diagnosis and treatment of autism spectrum disorders” (202007030002), the Guangdong Key Project in “Development of new tools for diagnosis and treatment of Autism” (2018B030335001), and the Open Project Fund from Key Laboratory of Reproduction Regulation of NHC (No. KF2018-03) to set up the China Multi-center Preschool Autism Project (CMPAP) to address this challenge.

## Materials and Methods

### Overall Design of the China Multi-Center Autism Project

**Figure 1:**
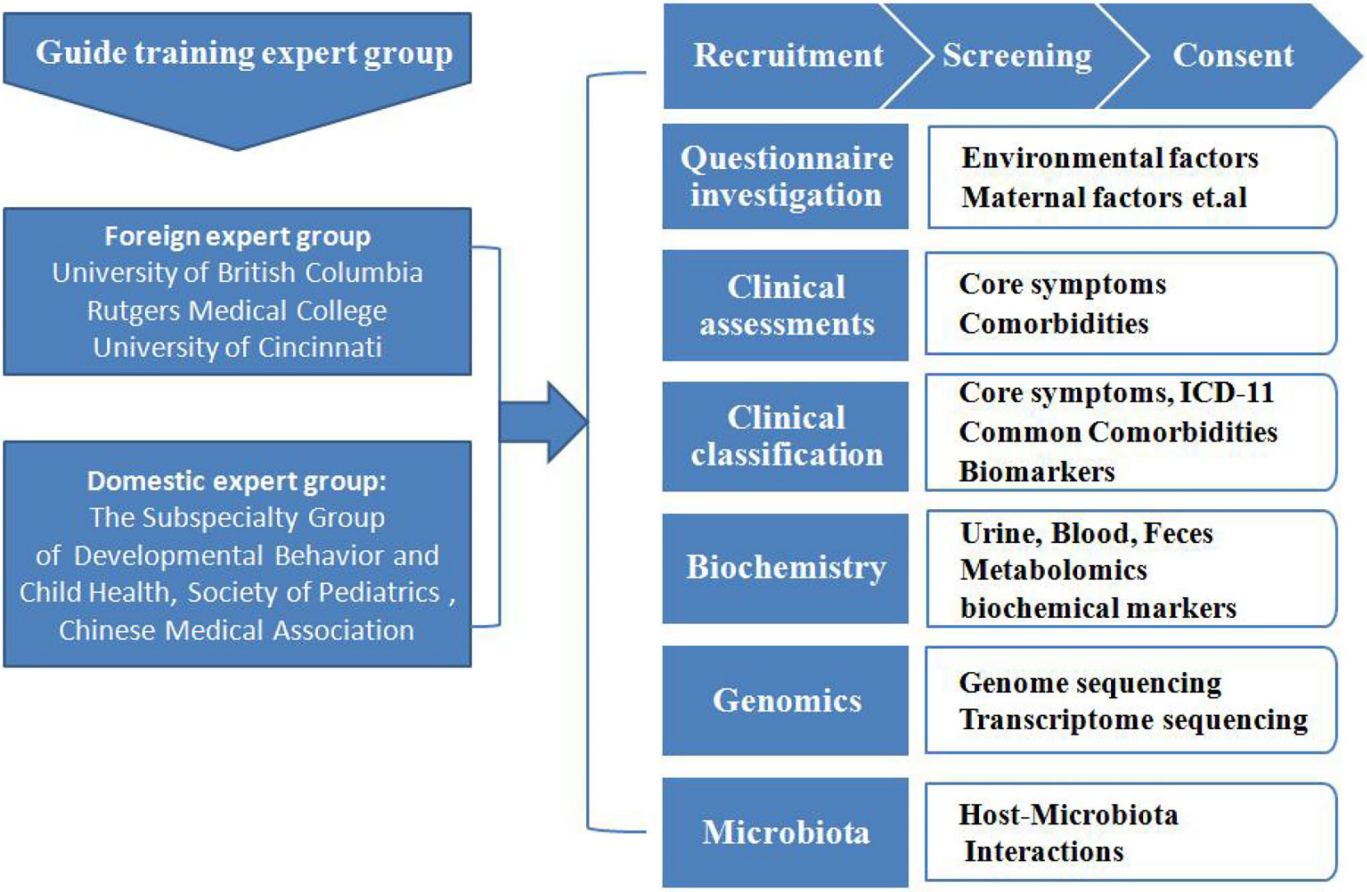
The recruitment and assessment procedures. The study population was selected from 13 cities in China. The diagnoses were made after a series of structured interviews were conducted by a psychologist and an experienced developmental pediatrician at the Children's Hospital, based on the criteria for autism defined in the Diagnostic and Statistical Manual of Mental Disorders, 5th Edition (DSM-5). The typically-developing (TD) controls were recruited from online volunteers to match the children with ASD in terms of age, gender, and their parents' levels of education. Participation in this research was voluntary, and parents who were willing to let their children participate in the study signed informed consent forms.

**Figure 2:**
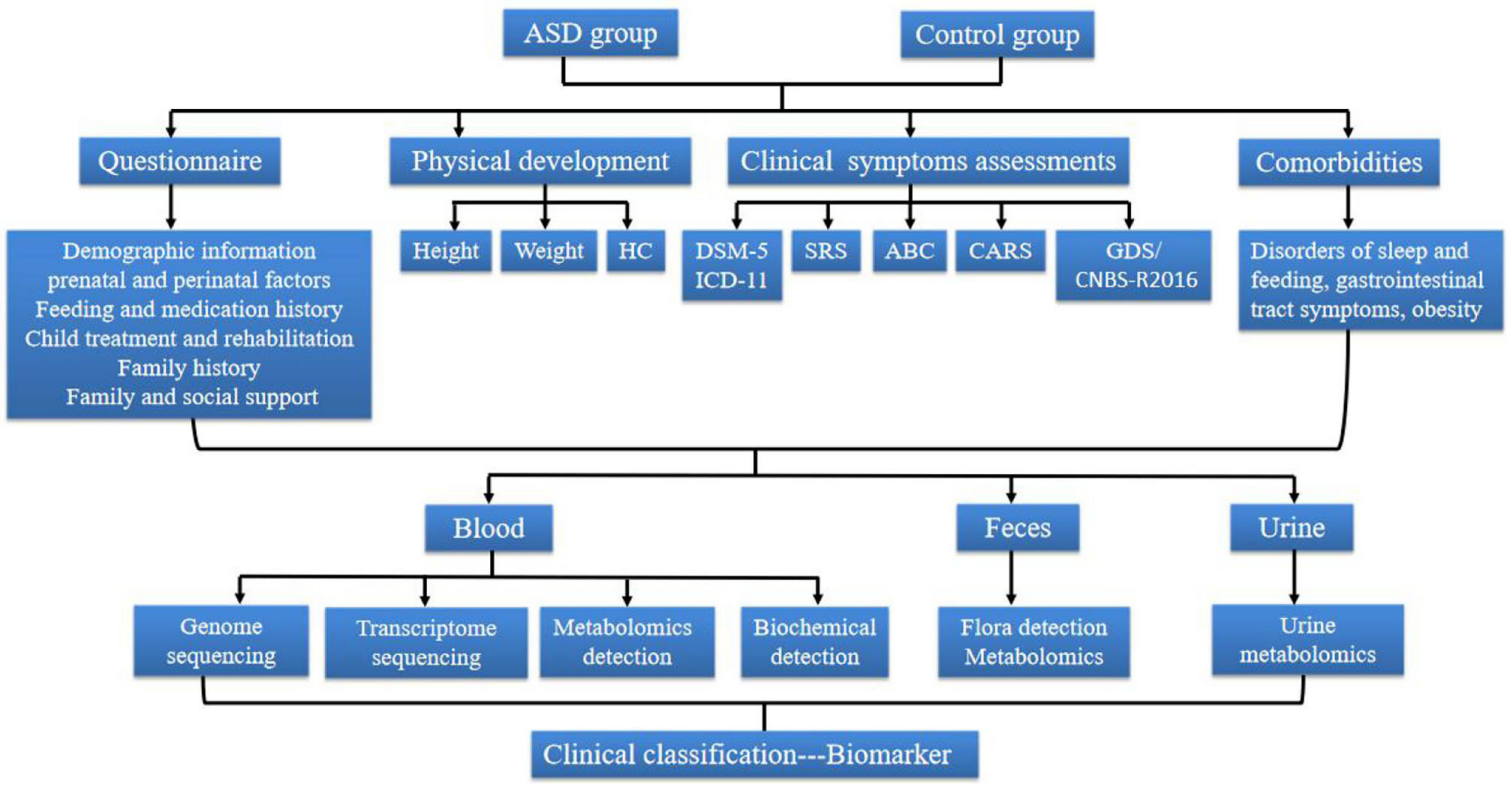
The Flow diagram of the study protocol. ASD, Autism spectrum disorder; HC, Head circumference; DSM-5, Diagnostic and Statistical Manual of Mental Disorders, 5th Edition; SRS, Social Responsiveness Scale; ABC, Autism Behavior Checklist; CARS, Childhood Autism Rating Scale; GDS, Gesell Developmental Scale; CNBS, Children Neuropsychological and Behavior Scale.

**Figure 3:**
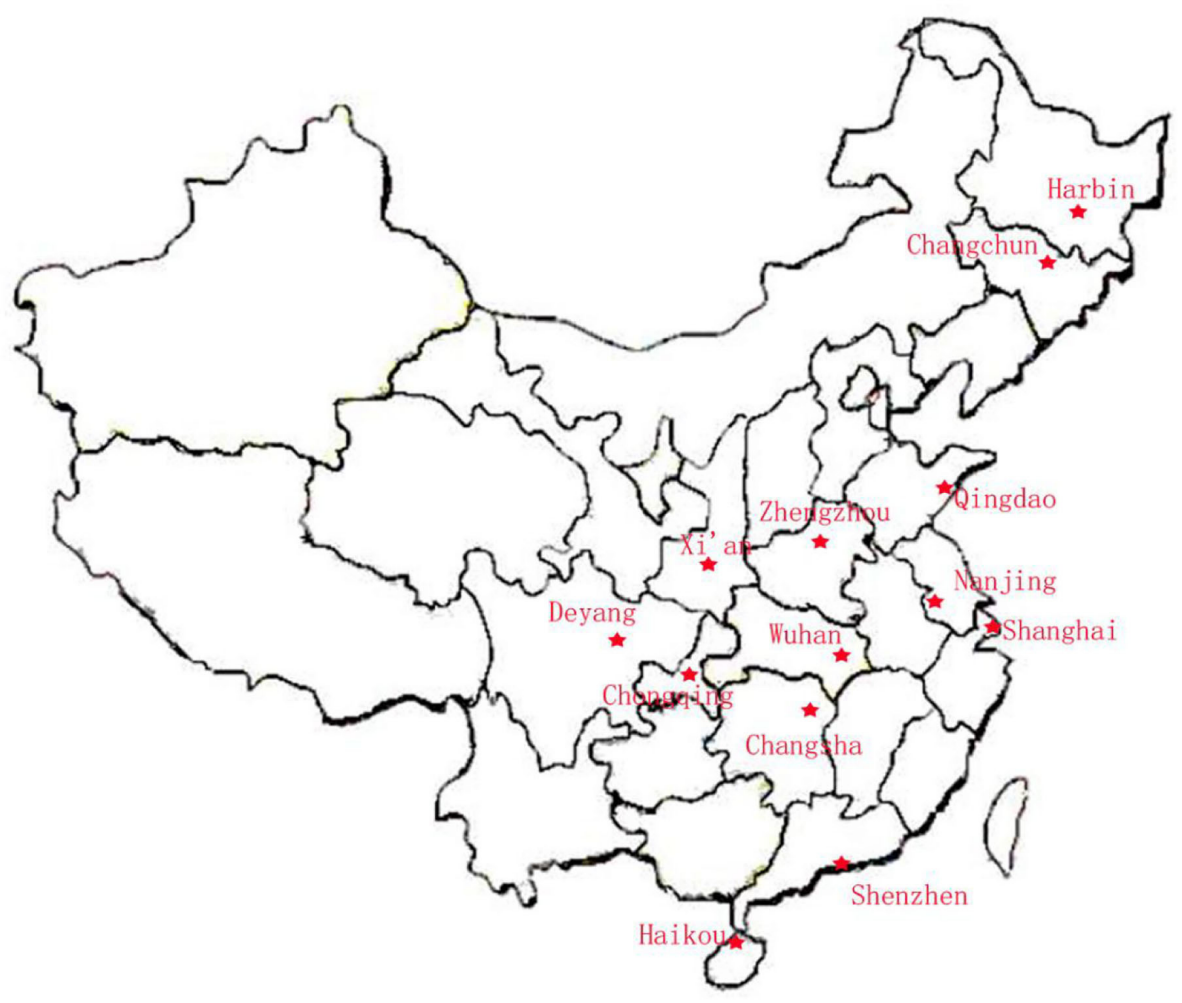
Location of the 13 cities in the CAMP study. The study population was selected from 13 cities in the North, East, West, South, and Middle of China: North = Harbin, Qingdao, and Changchun; East = Shanghai and Nanjing; West = Chongqing, Deyang, and Xi'an; South = Shenzhen, Haikou, and Changsha; and Middle = Wuhan and Zhengzhou.

**Figure 4:**
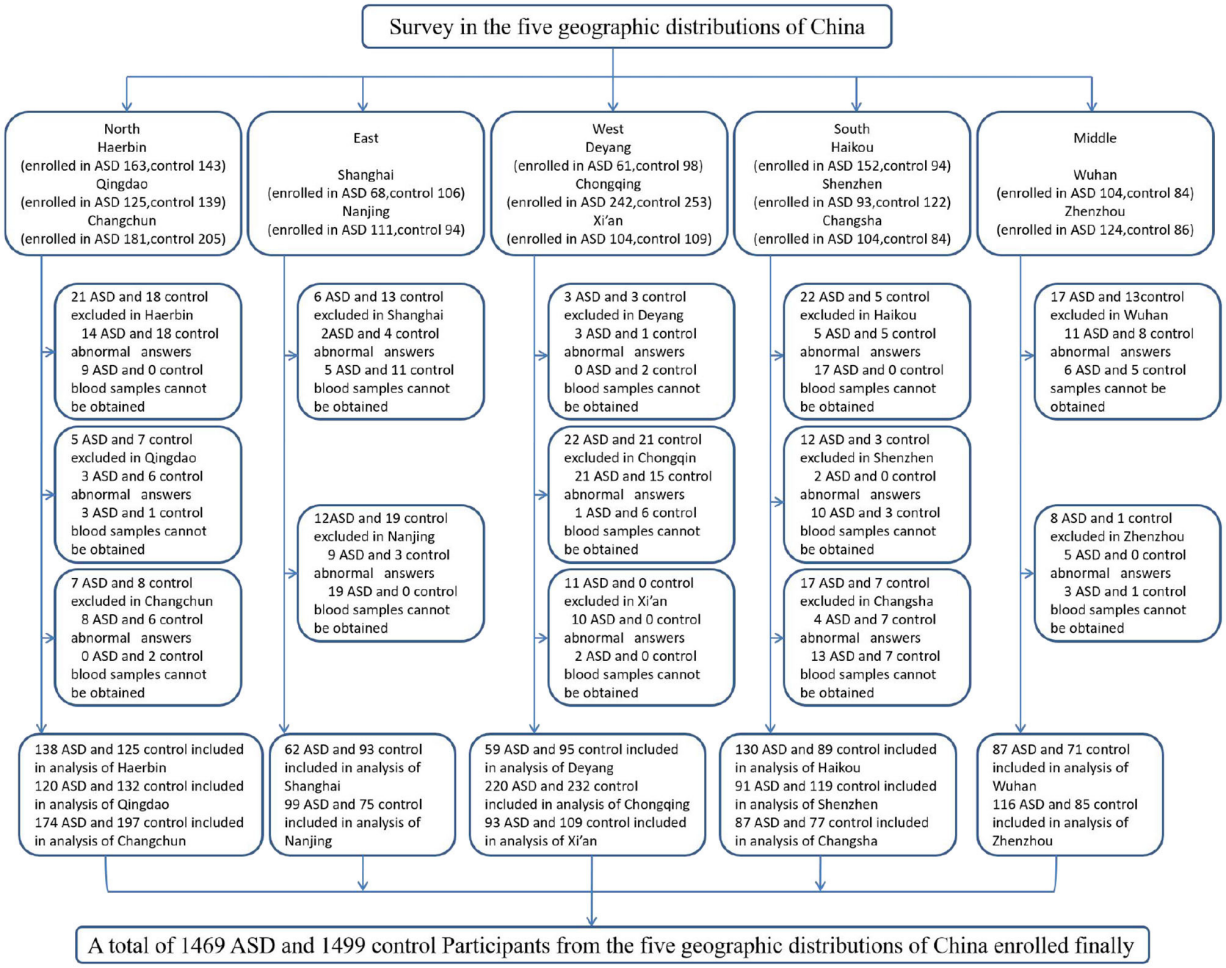
The sample screening flowchart of the study protocol.

The CMPAP is a case-control study that included nearly 3,000 participants (1,469 preschool children with ASD and 1,499 typically-developing(TD) controls) from 13 cities in China from May 2018 to December 2019. The project was prospectively sponsored by the Subspecialty Group of Developmental and Behavioral Pediatrics, the Society of Pediatrics, and the Chinese Medical Association, with the participation of 13 members of the China Autism Clinical Research Alliance. An overview of the study protocol is given in Figures 1, 2. An overview of the flow diagram, protocol, recruitment and study procedures, and sample screening flowchart are given in Figures 1–4.

### Participant Selection Criteria

Preschool children were selected from outpatients and children in special education institutions in order to investigate the early occurrence of ASD. The ability to diagnose and treat autism is not comparable across China because of its vast territory and scattered population, and is very difficult to conduct stratified random cluster sampling across the country. Therefore, the study population was selected from 13 cities in the North, East, West, South, and Middle of China: North = Haerbin, Qingdao, and Changchun;' East = Shanghai and Nanjing; West = Chongqing, Deyang, and Xi'an; South = Shenzhen, Haikou, and Changsha; and Middle = Wuhan and Zhengzhou. The diagnoses were made after a series of structured interviews were conducted by a psychologist and an experienced developmental pediatrician at the Children's Hospital, based on the criteria for autism defined in the Diagnostic and Statistical Manual of Mental Disorders (14), 5th Edition (DSM-5). The exclusion criteria were as follows: (1) a history of other independent developmental disorders, psychiatric disorders, Rett syndrome or other serious congenital diseases (e.g., congenital heart disease or seizures), or an acute or chronic affective disease during the previous 3 months; and (2) any food or drug allergy. The typically-developing (TD) controls were recruited from online volunteers to match the children with ASD in terms of age, gender, and their parents' levels of education. The TD children had no history of motor or language impairments, or social developmental disorders, according to the reports of their parents and teachers. Participation in this research was voluntary, and parents who were willing to let their children participate in the study signed informed consent forms.

### Interview Information

Information about six thematic areas was collected through face-to-face structured interviews with parents that were conducted by investigators who had received comprehensive professional training. The first part of the interview asked questions about demographic variables, including the child's age and sex, household income, family structure, and parents' age and educational level. The second part collected information about maternal risk factors, including the mother's health status and history of illness and stressful life events before and during pregnancy, complications during pregnancy, exposure to harmful substances (i.e., metals/pesticides, alcohol/drugs) during pregnancy, mode of delivery, medication during pregnancy, and use of supplemental vitamins/nutrients. The third part covered variables related to the child's feeding and medication, included feeding pattern, eating behavior, vitamin/nutrient supplements, and food allergies. The fourth part dealt with treatment and rehabilitation variables, such as the child's age at his/her first visit, age at diagnosis, length of rehabilitation training, and whether s/he had regressed. The fifth part obtained the family's psychiatric history and the sixth part collected data on social and family support variables, including the mode and amount of government subsidy, and the proportion of family income for rehabilitation training.

### Clinical Measures of ASD in Preschool Children

The diagnosis of ASD was confirmed using the DSM-5, and the symptoms of the patients were assessed with two parent-report instruments: the core symptoms of the Autism Behavior Checklist (ABC) (the score of normal children should be <53), the Social Responsiveness Scale (SRS) (the score of normal children should be <65) (15, 16), and one developmental pediatrician observation instruments: the Childhood Autism Rating Scale (CARS) (the score of normal children should be <30)(16). The neurodevelopment level of the children was evaluated with the revised Gesell Developmental Scale (GDS) (scores <75 indicate a developmental delay) or the revised Children Neuropsychological and Behavior Scale (CNBS-R2016), which has been revised and widely used in China (scores <70 indicate a developmental delay) (17). The most common comorbidities (i.e., sleep disturbances and gastrointestinal symptoms) of the patients were assessed using the Children's Sleep Habit Questionnaire (CSHQ) and the Gastrointestinal Severity Index (GSI).

### Biological Samples

We acquired blood, urine, and feces samples from the participants, which were collected according to standard procedures and stored immediately at low temperatures. The blood samples were used for biochemical, genomicand, and metabolomic analysis (11), the urine and fecal samples were used for metabolomic analysis, and the fecal samples were also used to detect intestinal flora (18).

### Biochemical Biomarkers

Alterations in the immune system (19), serotonin blood levels, and serum vitamin levels have been reported in ASD (20, 21). Increased serotonin blood levels are the most consistently replicated biochemical abnormality found in ASD (22), with ~27% of individuals showing significant elevations (23). Therefore, the levels of micronutrient, serotonin, and homocysteine in serum were determined.

### Genetic Markers

The blood samples were used to conduct whole-genome sequencing. Our aim was to identify the specific genes for different ASD subtypes that were based on the ICD-11, severity of core symptoms, co-occurring psychiatric symptoms, and developmental trajectories. The data were analyzed to try to improve the formulation of diagnoses and treatment plans for individual children with ASD.

### Environmental Risk Factors

Data on environmental risk factors were collected using questionnaires and electronic medical records. Increasing evidence indicates that environmental risk factors might play a larger role in ASD than previously assumed (24–26). Immune dysfunction has also been suspected in the prenatal development of ASD. That is why we collected the information about maternal risk factors (24) and potential protective factors (e.g., vitamin/nutrient supplements) (21, 27) that were described in the Interview section. Other related information included disease development, birth history, developmental history, past disease history, and family history of the children with ASD.

### Data Collection

We obtained four types of comprehensive information: (1) the interview data (see the Interview information section); (2) biochemical data (e.g., micronutrient, 5-HT, and homocysteine); (3) clinical evaluation data (DSM-5, ABC, SRS, CRAS, DQ, and common comorbidities); and (4) genomic, metabonomic, and intestinal flora data. All the participating units had to scan and upload the data, and the Affiliated Children's Hospital of Chongqing Medical University summarized the data and uploaded it to the database of the Chinese Clinical Trial Registry. Population characteristics were used to compare outcomes between the two groups.

### Statistical Analysis

Data were analyzed using SPSS version 19.0. Student's *t*-test was used for continuous data, and differences in the proportions of categorical variables were compared using the Chi-square test or Fisher‘s exact test. Binary logistic regression was used to screen risk factors and protective factors for ASD in the patients, and multiple linear regression was used to analyze the relationship between the core symptoms of children with ASD and vitamin A, vitamin D, folate, homocysteine, and serotonin. We included center as a variable in our multivariate analysis. Multiple imputation was used to replace missing values for outcome variables due to loss to follow-up in the two groups. *P* < 0.05 will be regarded as statistically significant.

## Discussion

The present project is a large-scale case-control study (ASD = 1,469, TD = 1,499) of autism, which was conducted in 13 cities in China, to examine age at diagnosis and treatment, family and social support, clinical core symptoms, common comorbidities, and gene and environmental factors. The children with ASD were classified according to the ICD-11, based on clinical core symptoms and comorbidities. Then, the diversity of flora, genomics, and urine and feces metabolomics were analyzed, in the hope of finding specific biomarkers of different subtypes of ASD using a large sample from multiple centers.

Early diagnosis and family and social support for children with ASD are very important for prognosis. The current diagnosis of ASD is limited because it is based on behavioral signs, so early diagnostics are difficult and subjective. The mean age at diagnosis has consistently been found to be 4–5 years (28). Parenting a child with ASD is very stressful, especially during times when the child needs treatment, and social support for parents and their child have been shown to be very important for successful family adaptation (29). Indeed, it has been reported that the quality of life of adults with ASD is more closely associated to the presence of family and social support than to the symptoms of ASD. Yet, there is still a lack of nationwide data in China about age at diagnosis and social and family support. The present study provides basic information about age at diagnosis and treatment and current family and social support for children with ASD in China, which can be helpful for making an early diagnosis and for developing individual treatment programs for these children.

The diagnosis of ASD has undergone considerable evolution in the past several years. Diagnostic evaluation involves a multi-disciplinary team of doctors, usually including a pediatrician, psychologist, and rehabilitation therapist (10, 30). The current diagnostic system is based on the DSM-5, but the ICD-11 represents the most up-to-date general medical system in the world (31). The ICD-11 is an authoritative reference for medical professionals to use for the diagnosis and treatment of physical and mental disorders. The system is dynamic and changes are made as more is learned about neurodevelopmental disorders and their underlying etiology, which enables more comprehensive assessments of patients' clinical manifestations and comorbidities (31). The ICD-11 divides ASD into 6 subtypes based on intelligence and language levels, which is conducive to more accurate clinical interventions. Many researchers have tried to discover new biomarkers for ASD, including genetic, transcriptomic, proteomic, metabolomic, and radiological changes, but there are no generally accepted biomarkers (11). As there has been no large-scale multi-center study of children with ASD using the ICD-11 classification, we speculate that different genotypes may have different sensitivities to being triggered by environmental factors, and have different clinical phenotypes. Thus, we should start with different clinical phenotypes to study the effects of different genes, biomarkers, metabonomics, and environmental factors. The present multi-center study not only collected data about environment risk factors, but it also collected blood, urine, and fecal samples to analyze genomics, transcriptomics, metabonomics, microbial diversity, serotonin, micronutrients, and mineral levels.

Hence, we speculate that combined with clinical manifestations, analyses of key expressed genes and metabonomics of children with ASD may provide a more accurate clinical typing system and new biomarkers for specific clinical subtypes. However, the present study still has some limitations. Despite it being a multi-center study in 13 cities, the effective sample size of the individual cities was limited by the low prevalence rate of ASD. In addition, this study did not include a longitudinal follow-up cohort. Moreover, some of the data are retrospective and may be affected by recall bias. Therefore, further cohort studies should be conducted using larger samples from multiple centers. Finally, due to the large sample size and the fact that few assessors in China are qualified to conduct the ADOS or ADIR, we did not use them for diagnosing ASD.

### Conclusion

Based on population data from 13 cities, in what is to our knowledge the largest case-control study of ASD in China, to date, the findings should help to: (1) assess the status of early diagnosis and intervention, social and family support, and clinical symptoms in Chinese children with ASD; (2) develop more accurate clinical subtypes and interventions based on the ICD-11; and (3) find new biomarkers for specific clinical subtypes of ASD.

## Ethics Statement

The studies involving human participants were reviewed and approved by The study was approved by the ethics committee of the Children's Hospital of Chongqing Medical University, Approval Number: (2018) IRB (STUDY) NO. 121, and registered in the Chinese Clinical Trial Registry (Registration number: ChiCTR2000031194). Written informed consent to participate in this study was provided by the participants' legal guardian/next of kin.

## Author Contributions

TY conducted data collection and analysis and drafted and revised the manuscript. JZ and QL supervised the collection and analysis of ASD patient samples. TL and JC conceived and designed the research, performed data analysis. LC, L-JW, F-YJ, YH, X-YK, M-JY, and C-HJ performed data analysis and interpretation, revised the article, and conducted general supervision. All authors contributed to the article and approved the submitted version.

## Conflict of Interest

The authors declare that the research was conducted in the absence of any commercial or financial relationships that could be construed as a potential conflict of interest.
